# The first comprehensive population size estimations for the highly endangered largest diving beetle *Dytiscus latissimus* in Europe

**DOI:** 10.1038/s41598-023-36242-w

**Published:** 2023-06-15

**Authors:** M. Balalaikins, G. Schmidt, K. Aksjuta, L. Hendrich, K. Kairišs, K. Sokolovskis, U. Valainis, M. Zolovs, M. Nitcis

**Affiliations:** 1 DU Nature Studies and Environmental Education Centre, Vienības Str. 13, Daugavpils, 5401 Latvia; 2 Institute of Life Sciences and Technology, Daugavpils University, Coleopterological Research Center, Parades Str. 1a, Daugavpils, 5401 Latvia; 3 Independent researcher, Heidelberg, Germany; 4 Department of Entomology, SNSB-Zoologische Staatssammlung München, München, Germany; 5 Department of Biology, Lund University, Sölvegatan 37, 223 62 Lund, Sweden; 6 Riga Stradins University, Statistics Unit, Balozu Str. 14, Rīga, 1048 LV Latvia

**Keywords:** Biodiversity, Freshwater ecology, Zoology

## Abstract

*Dytiscus latissimus* (Coleoptera Dytiscidae) is an endangered diving beetle throughout its range. It is one of the two species of Dytiscidae listed in Annex II of the Habitats Directive, IUCN red list and in many national level legislations and therefore strictly protected. The conservation of endangered species first of all requires an assessment of their population size. Until now, a method has not been developed for estimating the size of *D*. *latissimus* populations. The article summarizes the results of two studies carried out independently in Germany and Latvia. Both studies were carried out in one water body used recapture method but with a different spatial placement of traps, which, according to our data, is an important factor in population estimation. We evaluated Jolly-Seber and Schnabel approaches of estimating aquatic beetle's populations and found that confidence intervals obtained by different methods in our research do not differ significantly, but combination of both models provide the most accurate estimates of population dynamics. As part of the study, we concluded that the populations of *Dytiscus latissimus* are relatively closed, so we accept that the Schnabel estimate shows more accurate data. By fixing the places of capture of each individual, it was found that females live mainly locally, and males actively move within the water body. This aspect indicates the advantage of the spatial placement of traps compared to the use of transects. The results of our study show a significantly higher number of both captured and recaptured males Such a sex ratio may indicate both a greater activity of males and differences in the sex ratio in the population. The study confirmed that environmental changes, such as the water level in a water body, can also significantly affect the result of a population assessment. In the frame of *D. latissimus* monitoring, to obtain an objective estimation of the species population size we recommend using four traps for each 100 m of water body shoreline with 4–8 censuses, dependently on the recapture rate.

## Introduction

Dytiscidae is the most diverse beetle family that inhabits freshwater environments. The family contains more than 4200 known species that are divided in 10 or 11 tribes with predicted total species richness of around 5400 species^1–4^. Species of the Dytiscidae family inhabit a broad range of habitats from large lakes to seasonally drying ponds, peatlands and salt marshes, mountain and lowland ponds which can be oligotrophic to eutrophic^5–7^. Despite the fact that there is an increase in taxonomic, systematic and phylogeographic studies dytiscids are still an understudied group in terms of their ecology and biology^8^. One of such understudied species that attracts more attention due to its inclusion in several legislative acts in Europe is *Dytiscus latissimus*, with 44 mm body length one of the largest diving beetle species in the world (Figure B). *D. latissimus* is western Palearctic species with relatively large distribution range, occurs in most central European countries from France and Italy in the south, Sweden and Finland in the north, ranging eastwards to Western Siberia^9^. It inhabits a wide variety of freshwater environments such as shorelines of dystrophic and mesotrophic lakes that are densely vegetated by *Carex* and *Equisetum*^6^ or surrounded by flooded *Phragmites* belts^10,11^. In the south of its distribution, *D. latissimus* prefers to inhabit more oligotrophic lakes and in north it occurs mainly in the most nutrient-rich lakes^12^. Three of the most important factors necessary for *D. latissimus* are (1) the permanency of the water body that covers at least 1 ha and is at least 1 m deep (2) the specific plants along the shoreline necessary for oviposition (*Caltha palustris, Carex acuta, Carex pseudocyperus, Carex rostrata, Menyanthes trifoliata*) and (3) sufficient food supply for larvae, which is mainly formed by caddisflies Limnephilidae (Trichoptera) larvae^6,12–14^.

The populations of *D. latissimus* have been declining over most of its range since the early twentieth century, especially in the western part, while Latvia has the largest number of confirmed localities in the Baltic states^6,15^. In Germany *D. latissimus* population trends indicate a rapid decline since the 1960s^16^. At the beginning of the twentieth century the species occurred in most of the territories in Germany but after 2000 it has been observed only in five out of 16 federal states of the country, with stable populations only in the federal states of Brandenburg, Mecklenburg-Vorpommern and Bavaria^10,11,17,18^.

*D. latissimus* is included in the European Habitats Directive 82/43/EEC^19^ as well as in the Bern Convention Annex II, and it is also protected by legislation in many EU member states. The putative causes for the population decline are understudied and include drainage of lakes and agricultural land, agricultural intensification which contaminates freshwater systems with fertilizers and pesticides, urbanization, industrialization, deforestation, climate change and invasions of alien species^20^ or by increasing the abundance of more competitive species that prefer higher mean annual temperatures (e.g. the diving beetle *Cybister lateralimarginalis*)^11^. These factors may have an indirect effect on *D. latissimus*, contributing to the decline of the caddisfly population and thus the beetle larvae lack prey^21^.

Most of the research on *D. latissimus* is about distribution areas in different European countries, which has provided the basic knowledge about preferred habitats and possible factors of decline^22–26^. Studies on feeding preferences of *D. latissimus* suggest that during larval development they prefer Limnephilidae larvae, lack of which may influence the habitability of a water body^13,21^. Majority of the available data on the species currently is simple reports on presence/absence. Very little is known research on the population dynamics, rates of dispersion and spatial distribution within natural habitats has been done^27^. Given that estimating population sizes is a key in animal ecology, evolution, and conservation biology^28–31^ and that the assessment of the *D. latissimus* populations must be carried out according to European Union legislation, it is essential to develop an effective method. Due to a hidden lifestyle it is impossible to count all individuals in the population thus, the Capture-Mark-Recapture (CMR) method has been widely used by field biologists and ecologists to investigate the dynamics of biological populations^27,32–35^. There are various statistical models for analysing CMR data^35–38^, and methodologies for zoological field work^38–40^. To develop effective methods for specific groups of organisms, it is necessary to conduct studies that evaluate various approaches and consider the ecological characteristics of the target species, resulting in the most reliable data on the population size.

This is the first comprehensive study of the population biology of the critically endangered diving beetle *D. latissimus*, in which population size, density, age distribution and life expectancy were assessed. To evaluate the CMR approach in assessing the *D. latissimus* population size and the patterns of spatial distribution, we compared two independent extensive field surveys in different parts of its distribution range in the Northeast of Germany, in the nature reserve “Rothes Moor bei Wesenberg”^41^ and in the East of Latvia, in the nature protected territory "Rāznas Nacionālais Parks". In the analysis of the collected CMR data, we also compared two approaches, the Schnabel^42^ and Jolly Seber method^43,44^. Both studies used the same recapture method but differed in the spatial placement of traps. The aims of this study are to evaluate different approaches to estimating aquatic beetle populations using recapture method and describe movement patterns. Moreover, we provide recommendations for future monitoring programs.

## Methods

### Study area

**Figure 1:**
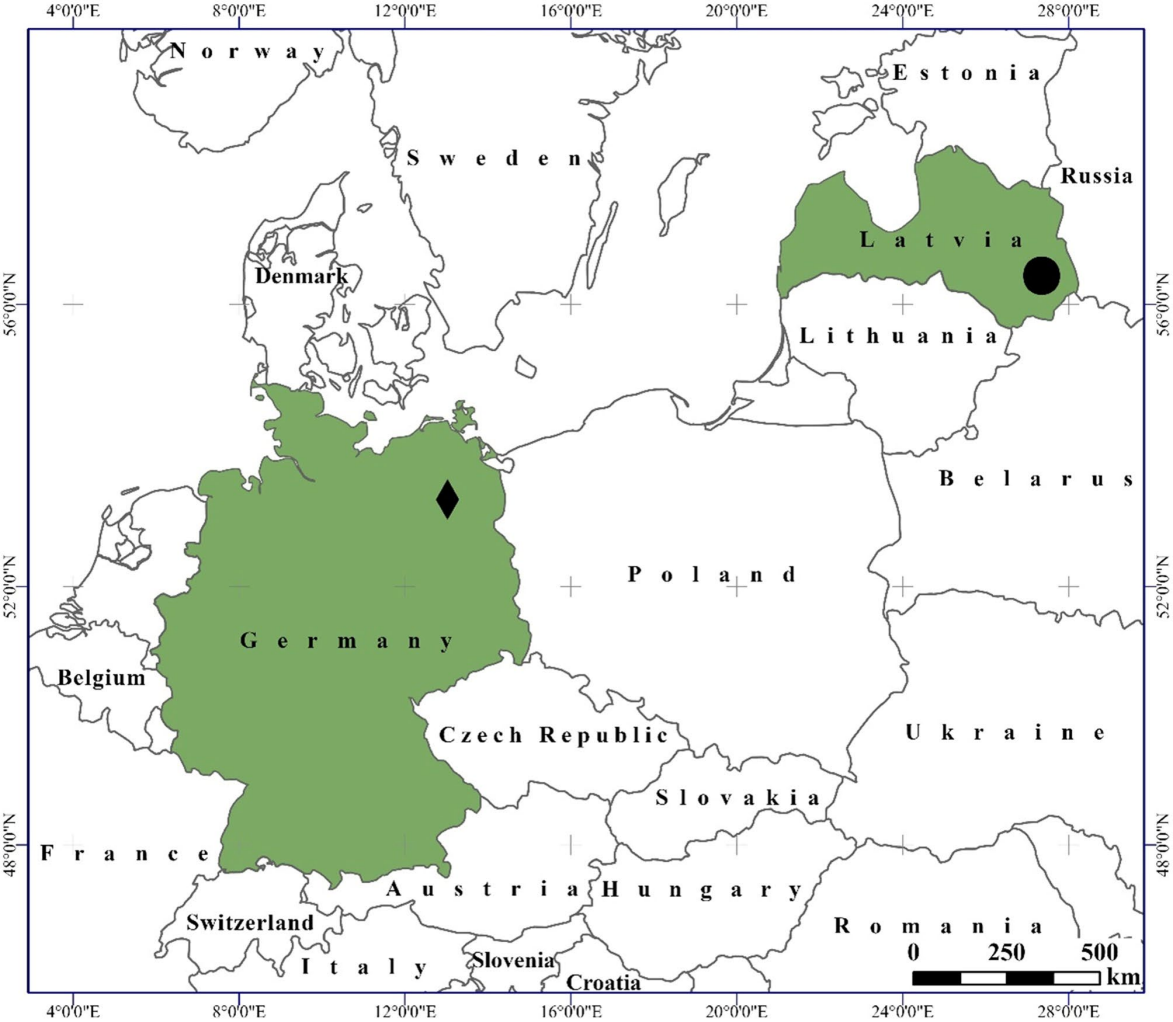
Location of study sites. The diamond shows the Lake Rothemoorsee in Germany and circle the Lake Glušonoks in Latvia.

The study area included two water bodies in which traps were set: The Lake Rothemoorsee (13.0253275°E 53.2774556°N) situated in the Northeast of Germany, in the nature reserve “Rothes Moor bei Wesenberg” and Lake Glušonoks (27,3,599,552°E 56,3,814,767°N) situated in the East of Latvia, in the nature protected area "Rāznas Nacionālais Parks" (Fig. 1).

**Figure 2:**
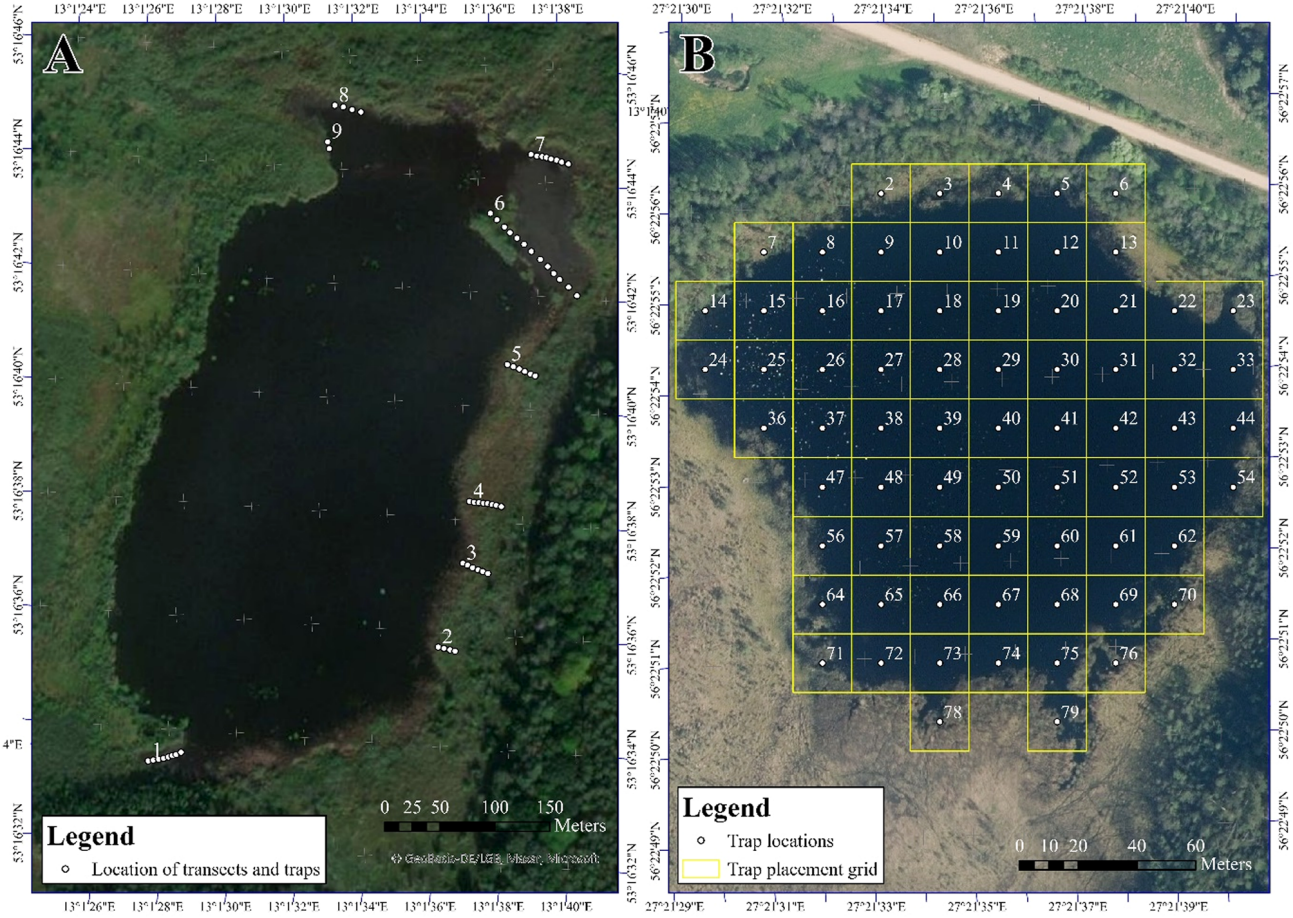
Spatial location of traps in research sites. (**A**) Lake Rothemoorsee in Mecklenburg-Vorpommern, Germany (**A**); Lake Glušonoks in Latvia (**B**). For the basemap used the wms service: ESRI World Imagery (Source: Esri, Maxar, Earthstar Geographics, and the GIS User Community). Source date: 13.09.2020. URL: https://services.arcgisonline.com/ArcGIS/rest/services/World_Imagery/MapServer".

**Figure 3:**
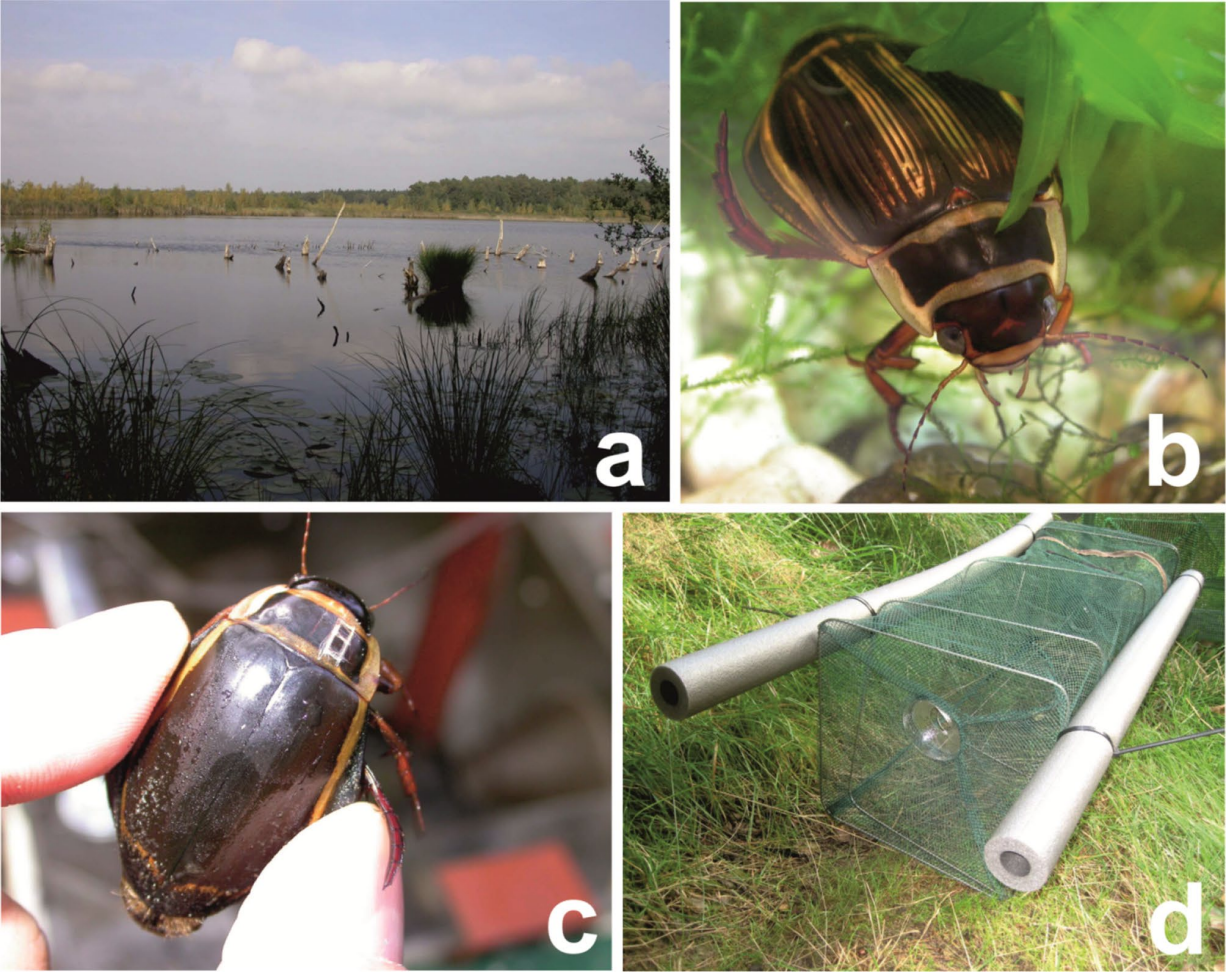
Habitat in the Lake Rothemoorsee (**a**), a female of *D. latissimus* (**b**), marked male of *D. latissimus* (**c**), and a trap used in Germany (**d**).

Lake Rothemoorsee (Figs. 2a, 3a) is a slightly dystrophic to mesotrophic lake deficient in lime, surrounded by large bogs and marshes of intermediate type with former peat layer in the bogs. Several species of sedges (*Carex*.spp.) are common in the shallow water zone of the lake; most parts of the lakeside strip are overgrown with dense stands of the common reed *Phragmites australis*. Near the lakeside the white lily (*Nymphaea alba*) is growing inside the stands of the common reed. In more open water the yellow water lily (*Nuphar lutea*) covers most part of the water surface. The lake is inhabited by predatory fish. Pike and perch were regularly caught in traps during the survey. Special studies of the ichthyofauna in the lake were not conducted, therefore, determining the population density of predatory species is not possible. according to the authors of the study, it can be considered that the population density of predatory fish in this lake is quite low. The Lake Glušonoks corresponds to the habitat included in the first Annex of the Habitats Directive: Natural eutrophic lakes with Magnopotamion or Hydrocharition type vegetation^19^. It is a brown-water (poly-humic) lake with diverse vegetation. Several species of sedges (*Carex* spp.) are common in the coastal zone of the lake; part of the coastal strip is overgrown with common reeds. Near the shore, the water soldiers (*Stratiotes aloides*) and the yellow water lily constitute most of the surface coverage. No predatory fish were found in the lake during the study period, so the presence of predatory fish during this period is unlikely. However, in the course of later research, one specimen of pike was found in the lake. We assume that it got into the lake after the research, but the existence of a very small population of pike in the lake is not excluded.

The average depth of Lake Rothemoorsee is 0.4 to 1.5 m and the Glušonoks is 1 to 1.5 m., The Lake Rothemoorsee has an area approximately twice the size of the Glušonoks 5.5 ha and 2.4 ha respectively.

### Trap construction and placement in Rothemoorsee

In the Lake Rothemoorsee nine line transects were selected along shallow shores with dense macrophyte growths (Fig. 2a). The transects were calibrated and marked on site. The traps within the transects were placed 3 to 5 m apart. Due to fluctuations in the water level, the number of traps varied between the capture sessions and study years, the exact locations of the traps were slightly adjusted. The maximum number of traps used on the nine transects was 51, but minimum 21; the traps were removed in November. Based on the assessment of lakeside the water level was measured in ordinal scale with five categories: very low (water level significantly below the edge of the shore), low (water level slightly below the edge of the shore), moderate (water level in the same line with the edge of the shore), high (water level slightly above the edge of the shore) and very high (water level significantly above the edge of the shore).

Large (55 × 23 × 23 cm) traps, made of plastic mesh and a metal frame, were baited with bloody pig liver (Fig. 3d). Traps had self-made plastic funnel entrances at both ends with a funnel opening of 30 mm. Two floats were attached along the sides of the traps so that the top was kept above the water level and the captured individuals could breathe. The traps were removed 48 h after being set up. In the shallow water zone, they were located at the bottom, further and deeper in the lake they were floating partly beneath the water surface.

### Trap construction and placement in Lake Glušonoks

**Figure 4:**
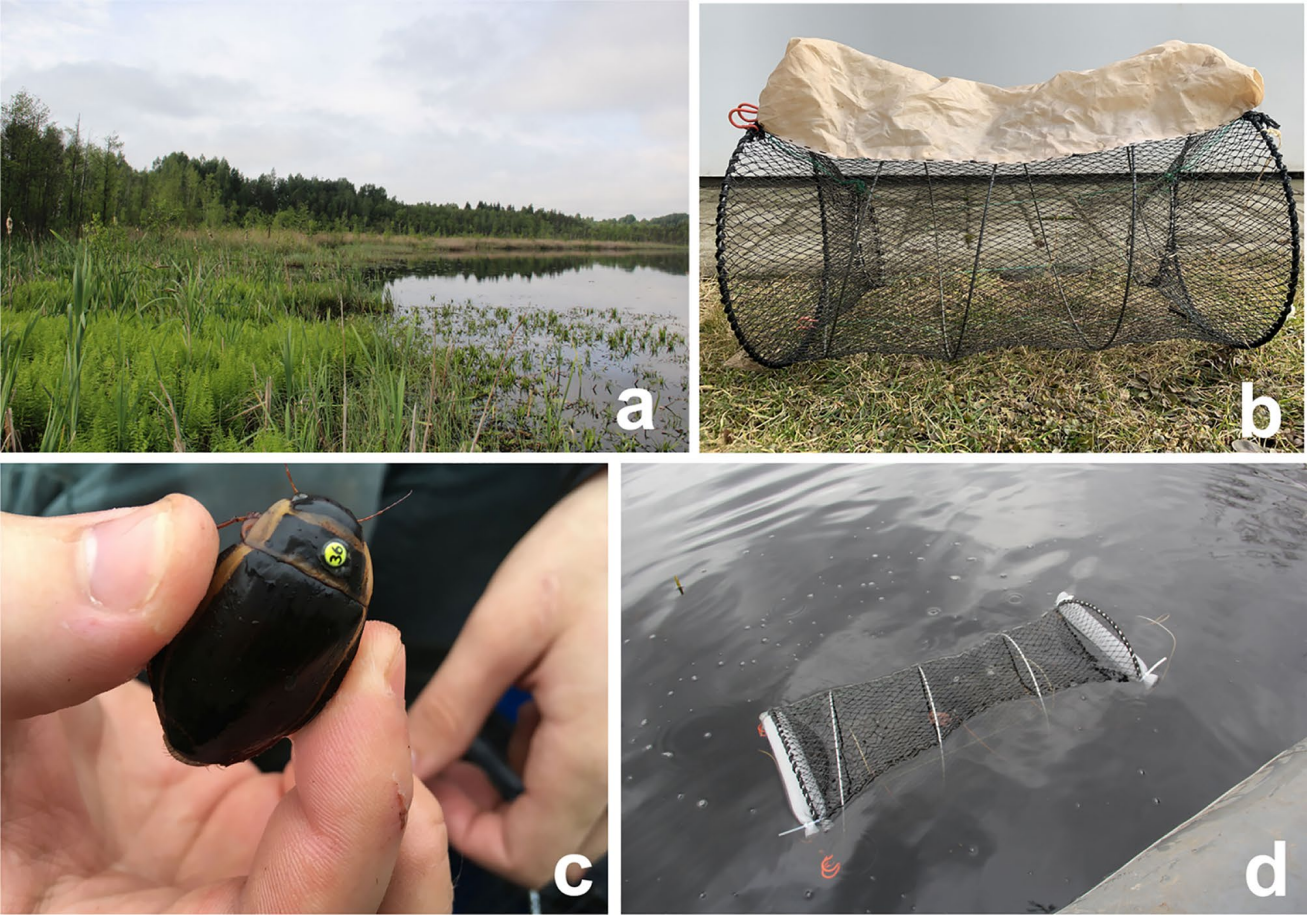
Habitat in Latvia, Lake Glušonoks (**a**), a trap set on the lake bed (**b**), traps used inthe Lake Glušonoks: marked male of *D. latissimus* (**c**) a trap set near the water surface (**d**).

The surface of Lake Glušonoks (Fig. 4a) was divided into 25 × 25 m quadrants (Fig. 2b). Two paired traps were set in the centre of each quadrant one slightly floating on top of the surface and one on the lakebed. Considering that both traps formed a single trapping unit we did not distinguish whether beetle was caught in the bottom or top trap in the analysis. We used cylindrical baitfish traps covered with netting. The square lattices of the net measured 1 × 1 cm. Trap height was 55 cm, diameter 32 cm. Each trap had circular entrance with 12 cm diameter. Sealed foam tubes were attached to the top traps so they would float and have oxygen for the beetles (Fig. 4d). Traps set on the lakebed were covered with waterproof fabric which was filled with atmospheric air when being submerged (Fig. 4b). In order to keep the bottom traps on the lakebed we attached two 2 kg weights to them. Both traps were connected by a rope at both ends of the cylinder which in order to anchor the trap near the water surface from drifting outside of the quadrant. We used fresh cattle liver as bait. Traps were removed 48 h after being set up.

### Timing of data collection

Data acquisition in the Lake Rothemoorsee was carried out in three periods, during which the traps were set up once a month: May–November 2009 (eight capture sessions, 20–39 traps per session), March–November 2010 (10 sessions, 41–51 traps per session), April–November 2011 (nine sessions, 38–46 traps per session, in November only 21 traps). Field surveys in Lake Glušonoks were done from April 2018 to November 2018 with eight surveying trips with approximately one-month intervals. In total 71 trap systems were used for each survey.

### Capture-Mark-Recapture (CMR) technique

The CMR methodology in both studies followed similar principles, assuring that so the implementation did not differ significantly. Each captured *D. latissimus* was marked with a unique, permanent number directly as the traps were emptied. The collection site, sex, time date, and notes, which may include additional information about the age and morphological characteristics of the specimen were documented for each marked individual. The specimens were released back into the same place where they were captured. The slight differences in the CMR methodology between the countries are described below.

In the Lake Rothemoorsee, the marking took place by engraving the pronotum (Fig. 3c), as tested by Mölle^45^ on a large number of *Dytiscus marginalis*. The engraving was performed with great caution with the help of a dental burr without damaging the animal. The marking was a continuous individual number for each year for each sex. The marking year was denoted by a special symbol. Freshly metamorphosed specimens with soft exoskeletons were not marked because the risk of injury was too high.

In the Lake Glušonoks Beetles were marked by fixing labels to the elytra with cyanoacrylate “Superglue”, following a modified method described by Davy-Bowker^46^. After removing the trap, each unlabelled specimen was marked. Each individual was then placed in a separate clean container without water. Shortly afterwards the specimen was dried using a paper towel and the surface of the prothorax was cleaned using a paper towel soaked in 96% ethanol. We used commercially available labels used to mark bee queens. These are made of plastic and are approximately 2 mm in diameter. Each contains three pieces of information: a unique two-digit number, colour of background and colour of the numbers. Once ethanol evaporated from the prothorax surface a label was prepared by applying a small amount of cyanoacrylate glue to the bottom. Label was applied on the pronotum of the beetle (Fig. 4c) and care was taken to avoid any of the glue getting on the sutures. After about five minutes of letting the glue dry, the specimen was placed back into a plastic container for further polymerization of the glue for additional 10 min. When the glue was completely polymerized the specimen was released in the same place where it was collected.

### Statistical analysis

**Figure 5:**
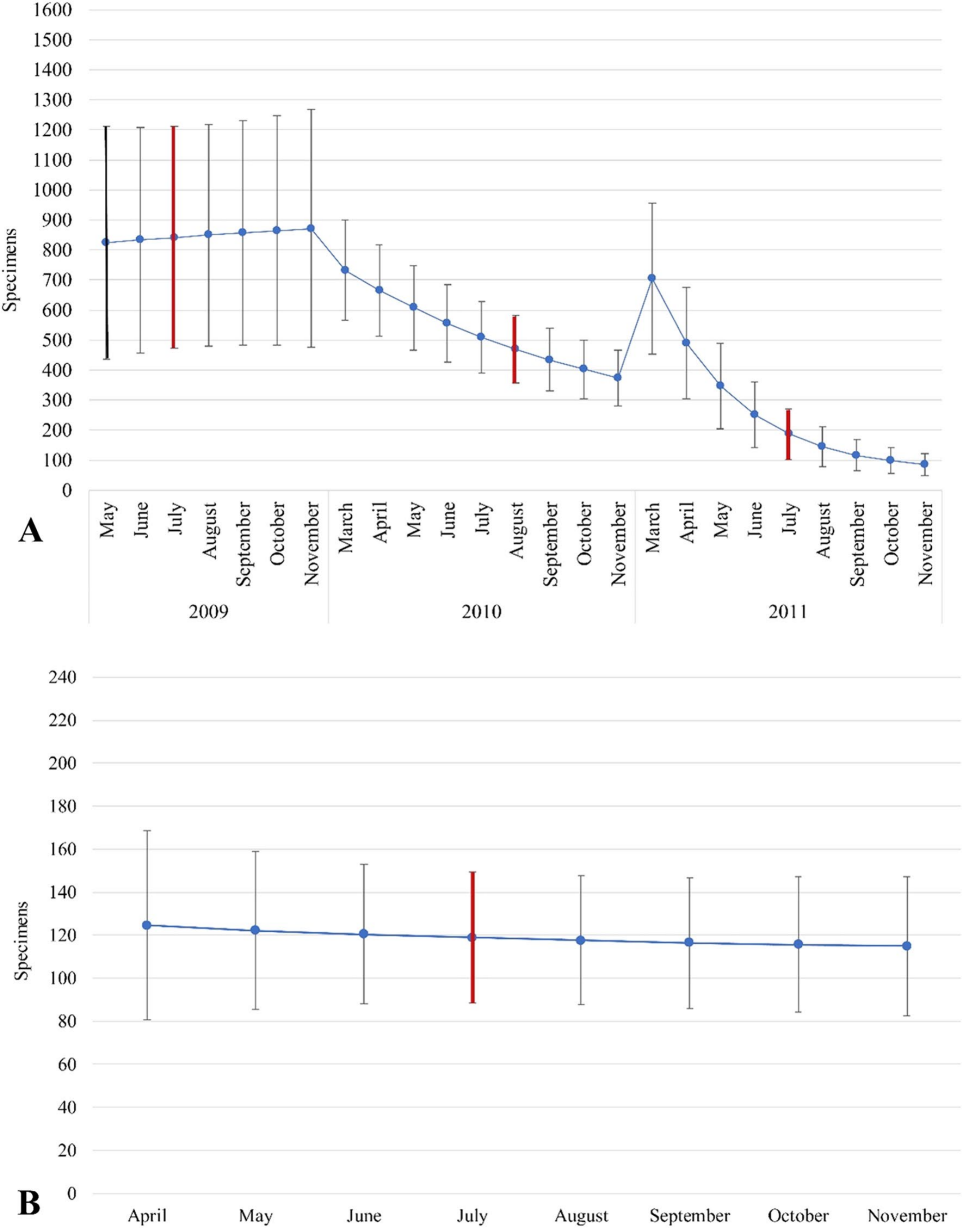
Jolly-Seber population size estimates with 95% confidence interval: Lake Rothemoorsee (**A**) Lake Glušonok (**B**). A bar coloured red indicates the emergence period of young imago.

Firstly, descriptive statistics were calculated: (1) total number of trap checks, beetles per trap, captures, marked and first captured, and recaptured of marked specimens every year (Table 1); (2) the centre point (median) and spread of captured and recaptured individuals (interquartile range) (Table 2); (3) the Schnabel estimate for closed populations (Table 2) and the Jolly-Seber model for open populations with a 95% confidence intervals (Fig. 5).

**Table 1:** Number of trap checks, beetles per trap, captures, marked and first captured, and recaptured of marked specimens every year.

Country	Year	Number of captures	Number of marked individuals every year	Number of recaptured of marked individuals every year	First capture of marked individuals of previous years	Recapture of marked individuals of previous years	Number of trap checks in the year	Beetles per trap
Germany	2009	407	344	60	2	1	227	1.80
Germany	2010	549	289	104	103	53	405	1.36
Germany	2011	237	101	17	101	18	335	0.71
Latvia	2018	239	92	63	–	–	568	0.42

**Table 2:** Descriptive statistics and Schnabel population size estimates.

Country	Year	Population size		Captured			Recaptured		
		Schnabel estimate	95% confidence interval	Min–max	Median	IQR (Q1–Q3)	Min–max	Median	IQR (Q1–Q3)
Germany	2009	1018	775–1483	23–107	59	34–75	0–16	9	2–16
Germany	2010	679	573–832	19–115	66	25–81	0–45	14	7–29
Germany	2011	563	471–705	1–91	14	13–39	0–10	4	1.5–5
Latvia	2018	208	167–276	20–43	28	21–40	0–30	9	2–19

*IQR* interquartile range, *Q1* the first quartile, *Q3* the third quartile.

**Figure 6:**
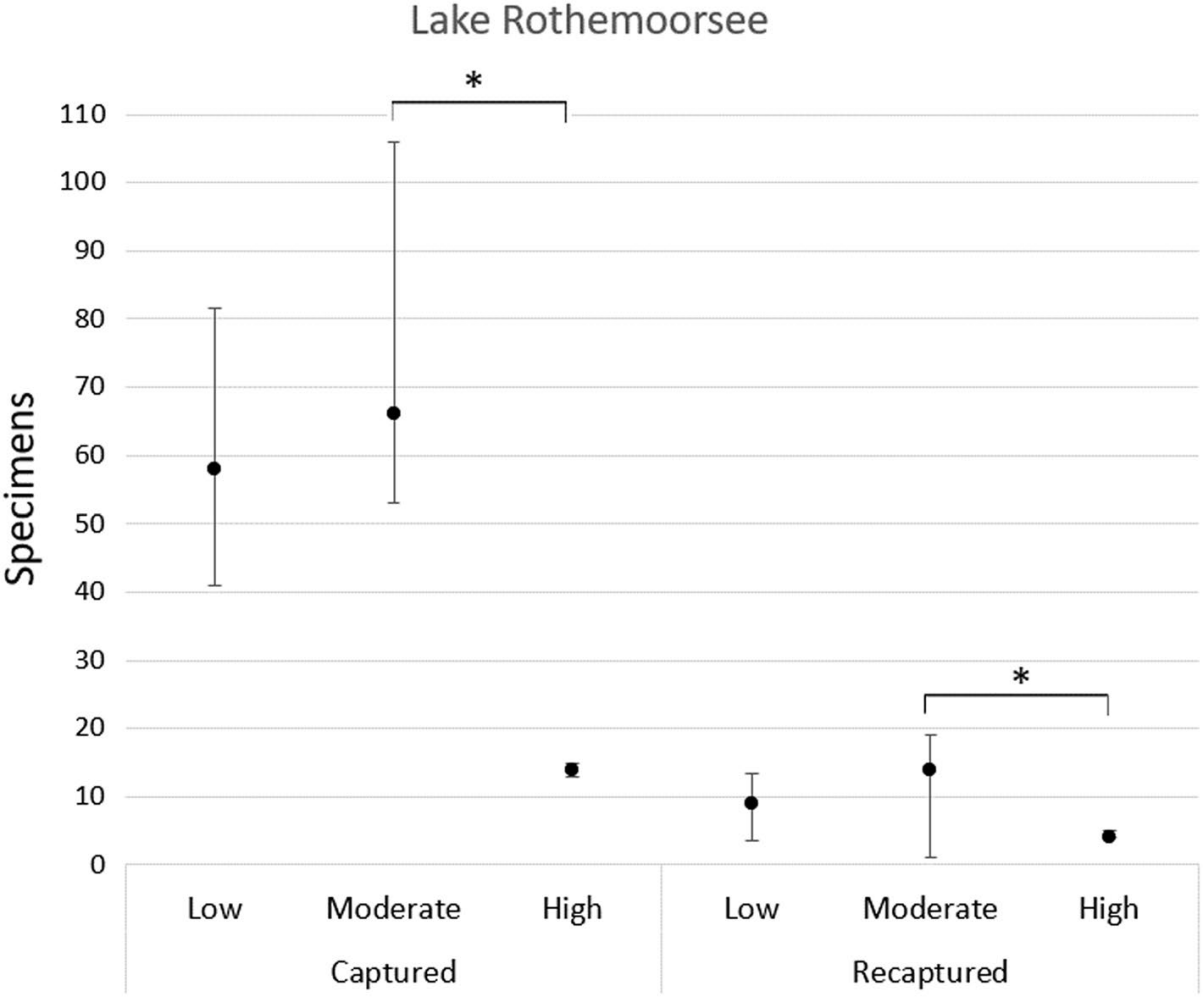
Difference of *Dytiscus latissimus* occurrence between water levels*.* Median with interquartile range (Q1–Q3) connected and marked with an asterisk show significant differences between water levels.

Secondly, to test statistical hypotheses the inferential statistics were calculated. To compare the number of captured and recaptured individuals between water levels the Kruskal – Wallis H test with Bonferroni post hoc correction was conducted for Germany (Fig. 6).

To determine the strength and direction of the association between water level and captured and recaptured individuals the Spearman's correlation was calculated. To develop recommendations for the minimum required number of traps for monitoring, we took advantage of the shortcoming of this study—the different number of traps. We used the confidence interval of population size as an indicator of the number of traps in order to find the optimal ratio of traps and the population size indicator, i.e., point where the confidence interval has the smallest size at the minimum number of traps.

As this observation study is replicated across seasons introducing random variable, the Generalized Linear Mixed Model (GLMM) was performed to identify factors influencing *Dytiscus* occurrence in natural habitat. Because the response variable (captured *Dytiscus* individuals) comes from the count distribution with overdispersion, the negative binomial distribution with the logarithmic link was applied. The sex ratio and the recapture individuals were used as explanatory variables. All possible models including the null model and interactions were calculated. To select the best model, the Akaike Information Criterion (AIC) was used. The GLMM was carried out separately for the countries because the number of traps in Latvia was the same throughout the study, but in Germany it varies between years. The number of traps was modelled as an offset. Statistical data analysis was conducted using the packages ‘fishmethods’^47^, marked, lmer and RMark implemented in R 4.0.4.^48^.

## Results

During three field seasons, 1193 specimens were captured in the Lake Rothemoorsee, and 239 were captured in the Lake Glušonoks during one season. Of all marked specimens, 181 were recaptured in the Lake Rothemoorsee and 63 in the Lake Glušonoks. Details of number of trap checks, beetles per trap, capture recapture events are appended in Table 1. Population estimates using the Schnabel approach are summarized in the Table 2, and using the Jolly-Seber (Fig. 5).

The results of GLMM showed that null model better explain captured individuals indicating no effect of explanatory variables (Supplementary Table S1). Significant fluctuations in lake water levels were observed during the study in the Lake Rothemoorsee. Spearman's correlation test showed a moderately significant negative association between the number of captured individuals and the water level in the lake (r_s_ =  − 0.486, *n* = 25, *p* = 0.014), indicating that the decline in the number of captured beetles could be correlated with the increased level of water in the lake; there was, however, no association between recaptured individuals and the level of water in the lake (*p* > 0.05). The Kruskal – Wallis H test showed significant difference of captured (X^2^(2) = 8.08, *p* = 0.018) and recaptured (X^2^(2) = 7.96, *p* = 0.019) individuals between water levels, where post hoc test revealed significant difference only between moderate and high water level (*p* < 0.05) (Fig. 6).

Based on data from recaptured individuals, the study estimated the distance between the points of the capture of individuals. The Mann -Whitney U test showed that the mean distance covered by recaptured specimens differed significantly between Latvia and Germany U = 1956.5, Z =  − 3.754, *p* < 0.001, r = 0.354 (medium effect size) (Table 3).

**Table 3:** Distance (metres) covered by recaptured animals.

Year	Country	Duration	Min	Max	Mean	SD	95% Confidence interval of the mean	CV, %
2009	Germany	1 month	5	390	156	117	106–205	75
		7 months	7	888	274	249	168–379	91
2010	Germany	1 month	2	382	154	100	129–178	65
		7 months	2	909	220	178	177–262	81
2011	Germany	1 month	36	273	177	99	101–253	56
		7 months	36	430	216	128	117–314	59
2018	Latvia	1 month	20	184	95	47	83–107	49
		7 months	20	523	169	107	142–196	63

*SD* standard deviation, *CV* coefficient of variation.

*Recaptured individuals in the same trap were not taken into account.

The multi-year trapping in the Lake Rothemoorsee allowed us to make inferences of the maximum lifespan of *D. latissimus* in the wild. One male (marked in October 2008) was found in April 2011. Therefore, it is reported here for the first time that the adults of *D. latissimus* can reach an age up to three years, – even in the wild.

## Discussion

We compared different approaches in estimating *D. latissimus* populations in two lakes in Latvia and Germany. Both studies were based on two main methods: trapping beetles with modified fish traps and the CMR method. These methods had been used in similar studies, on other organisms, however not on *D.latissimus*. Obtaining an accurate quantification of population size is often of prime importance in ecology and conservation biology^49,50^. CMR techniques have been widely used and specialized to study organisms throughout the field of biology^51^. This method is most suitable for obtaining reliable information on the number of individuals in populations of animal species with a hidden lifestyle. It has for example been successfully applied to estimate population size in the hermit beetle *Osmoderma eremita*^52^. In the present study, two approaches were used for CMR data analysis: the Schnabel method based on closed population model and the Jolly-Seber method based on open population model^43,44,53^. Combination of both models provide the most accurate estimates of population dynamics^54^.

The Schnabel method has been recognized as the most accurate in estimating freshwater crab^54^, and the Freshwater Anomura *Aegla longirostri*^55^ populations. The method provided results with relatively narrow confidence intervals, expectedly reflecting the real size of Orthoptera, Diptera and Hymenoptera populations^38^. The Jolly-Seber method is less efficient in assessing insect populations with potentially low recapture rate possibility, due to short-living stage of imago, lacking territorial behaviour and relatively high mobility^38^. Comparing the confidence intervals obtained by different methods in our research, the results do not differ significantly. This indicates that the populations of *Dytiscus latissimus* are relatively closed, most of the individuals lives in a limited space, and the proven life span of individuals reaches three years, which makes it possible to effectively use the CMR method. The significant difference lies in the population size estimates, Jolly-Seber estimator showing significantly lower population size results. Similar results, where the Jolly-Seber estimator showed significantly lower population size, were obtained when evaluating the population of *Psophus stridulus* (Orthoptera)^38^. It should be noted that this result is related to the low recapture rates, which is not true in our case. At the same time, for example, in Lake Glušonoks, based on the Jolly-Seber estimator data, more than 70% of the population was captured, which is objectively too optimistic. Based on these findings, we accept that the Schnabel estimate shows more accurate data, however, there are several additional factors to consider, which are presented in the next part of the discussion.

Methods of CMR analysis work particularly well for univoltine insects with clear spatial and temporal population boundaries, characterizing many habitat specialists. *Dytiscus latissimus* is a suitable species for use of the CMR method. In calculating the population size of a diving beetle species, a range of factors can influence the result. The first is that most dytiscid species disperse from one waterbody to another, primarily using aerial flight^12^. This process may be related to oogenesis flight syndrome^56^, characterizing female insect that migrate from their birthplaces before reproduction. Beetles’ migration can also be caused by changing conditions in the waterbody. Most previous studies were based on detecting flying insects, using light or flight traps^57,58^. However, using the light trap method it is impossible to draw conclusions about the rhythm of dispersal flight; thus, the CMR is the more effective method to determine flight intensity of aquatic insects^59^. For this purpose, the CMR method has been used on diving beetles by Davy-Bowker (2002), but that study did not estimate, population size^60^. Considering that no studies have been conducted to evaluate the intensity of dispersion of *D. latissimus* specimens, it is impossible to determine the effect of this factor on the results of population assessment. Another factor that can affect the estimate of the population size is life expectancy. Based on our results, *D. latissimus* can reach an age of up to three years. Accordingly, CMR allows obtaining additional estimates of mortality, life expectancy and maximum lifespan of individuals of the target species, which are key parameters characterising the dynamics of wildlife populations^61^. In case of *D. latissimus* several generations of beetles are included in the population estimation process.

According to the assessment of the abundance of *D. latissimus* populations in the Lake Rothemooresee and Lake Glušonoks, the number of individuals varies significantly. These differences are plausible, and can be explained by the facts that the Lake Glušonoks is just half of the size than Rothemooresee in Germany. Moreover, the shore regions of the the Lake Rothemoorsee are more richly structured and offer better protection for the larvae against predators (fishes) with their broad *Phragmites australis* reed beds that are flooded all year round. The analysis of various factors influencing the population size was not carried out within the framework of this study, which is laborious and impracticable in the framework of monitoring activities. The main difference between the methods applied in the Lake Rothemoorsee and the Lake Glušonoks was the placement of traps in the waterbody. In the Lake Rothemoorsee, the transect method was used and the traps covered only parts of the lake´s coastline, while in the Lake Glušonoks the grid method was used and the traps were evenly distributed throughout the lake. In both studies, the traps were placed in fixed locations, but given that the German study was conducted over several years, the results were influenced by the water level in the lake. For this reason, at different stages of the study, the number of traps differed.

Statistical methods were used to compare CMR data from both studies. A measure of spread (such as min–max, IQR, standard deviation etc.) shows how well measures of central tendency (such as mean, median or mode) represents the data^62^. The narrow range of IQR and min–max measures in the Latvian data (Table 1) indicates that the mean value describes the data better, and that the estimate of population size is more robust here than the corresponding results from the Lake Rothemoorsee. The range of 95% confidence interval of the Schnabel estimate from the Lake Rothemoorsee shows great variation among years of investigation. This suggests either a large effect of water level on population size, or a problem in the methodological approach. Special attention should be given to the results of 2009 where the range of 95% confidence interval was very high, indicating low predictive power of the Schnabel estimate. Finally, the Schnabel estimates for 2009 and 2011 differ by factor of 2, raising a question about the importance of the number and spatial arrangement of traps in the application of the CMR method. Certainly, the influence of water level may affect the population size, but the slight variations observed in Lake Rothemoorsee cannot be considered as the main factor affecting the population size of *D. latissimus*; therefore, we concluded that the changes in the number of individuals in different study periods are related to the spatial placement of traps.

Great effort has been done in the identification of ecological and methodological factors affecting detectability and estimation of the population size of various animal groups^63–67^. Since our work is pioneering the population estimates of *D. latissimus* many possibly confounding factors are yet to be understood. For instance, it still remains unknowns, since it remains unknown how effective the traps used in the study are and whether all the beetles attracted by the bait actually swim into the trap, and what is the risk of the beetles escaping from the trap.

Elevated wind speed has been identified as a factor influencing the recapture of *Culex tarsalis*^67^. Although diving beetles are primarily aquatic, wind speed can also affect these organisms. Wind induced waves affect the movement of both nutrients and various organisms in the water bodies, including diving beetles, which may affect recapturing success. Climatic conditions such as weather and atmospheric pressure can also have an effect.

All the above factors may bias population estimation. Thus, for a more accurate population assessment, that accounts for the influence of all factors, comprehensive models should be created, for example, in the^68^ or CAPTURE program. Future work may require Bayesian methods as they are increasingly used to analyse ecological data^69^ including spatial capture–recapture data sets^69^.

According to the results of the study in Lake Rothemoorsee, we have prepared a suggestion for counting *D. latissimus*, in the frame of EU monitoring. In order to be able to use the survey results in lakes of different sizes, we related the required number of traps to the length of the shoreline of the current body of water. The shoreline of Lake Rothemoorsee is 1200 m long, and according to our estimates the optimal number of traps for a water body with this shoreline length comprises 45–50 traps. Conversion of this result to 100 m of shoreline gives a ratio of 4 traps per 100 m, which can be used to plan the number of traps to monitor *D. latissimus*. This number of traps will provide the optimal ratio of data variation and the effort expended on setting the traps. In turn, the minimum number of traps should not be less than 35, as this greatly increases the range of the confidence interval. Estimators based on closed population model provided little fluctuating results with relatively narrow confidence interval after 4–8 censuses, dependently on the recapture rate^39^.

It seems that the number of traps per month plays a more significant role than the total number of traps per year. For example, it will be more effective to set 35 traps for 3 consecutive months (together 105 traps per year) than 21 traps for 5 consecutive months (together 105 traps per year). As water level plays a significant role in estimating population size, three consecutive years with less than 35 traps per month is not enough to achieve good monitoring results.

## Conclusions

*Dytiscus latissimus* is a species of high conservation value and the need to estimate its abundance is specified in European Union legislation. Prior to the present study, only a single publication on a small relict population in the Netherlands^27^ provided data for calculating the population size of *D. latissimus*. Our study provides a larger comprehensive framework for further studies aimed at monitoring and conservation of *D. latissimus* populations. This publication discusses two approaches to assessing populations of this species. In both studies, sufficient numbers of individuals were captured and recaptured to estimate population sizes, so the method used is appropriate for estimating populations of species. The comparison of data spread between years and countries suggests that setting shoreline traps only leads to overestimation, especially in lakes with an unstable hydrological regime, and needs to be improved to determine the population size more accurately. The absolute population size was determined with the method used in Latvia, which is more practical for smaller lakes, whereas in the larger lake in north-eastern Germany the activity density was determined.

## Supplementary Information

Supplementary Information.

## Data Availability

Currently data are available from the corresponding author on reasonable request. The datasets analysed during the current study are not publicly available yet because this data will be used to prepare at least one additional publication after which data will be publicly available.
