# Supplementary material for: The first comprehensive population size estimations for the highly endangered largest diving beetle Dytiscus latissimus in Europe
Source: Sci Rep. 2023 Jun 15;13:9715. doi: 10.1038/s41598-023-36242-w (PMC10272167; doi:10.1038/s41598-023-36242-w)
Supplement: Supplementary file 1 — Supplementary Information. [file 41598_2023_36242_MOESM1_ESM.docx]

Supplementary Information

Supplementary Table S1. Fixed effects in generalized linear mixed models to identify factors influencing *Dytiscus* occurrence in natural habitat. Data were analysed by negative binomial generalized linear mixed modelling including season as random effect variable.

| **Country** | **Model, (AIC)** | **Variables** | **Exp(B), 95% CI** | **Z value** | **P value** |
| --- | --- | --- | --- | --- | --- |
| Germany | 1 (237) | (Intercept) | 47.5 (34.4 – 65.7) | 23.4 | <0.001 |
|  |  |  |  |  |  |
|  | 2 (247) | (Intercept) | 46.7 (33.1 – 65.8) | 21.9 | <0.001 |
|  |  | Sex ratio | 0.97 (0.91 – 1.02) | -1.18 | 0.240 |
|  |  |  |  |  |  |
|  | 3 (248) | (Intercept) | 43.9 (23.8 – 80.7) | 12.1 | <0.001 |
|  |  | Recapture individuals | 1.05 (1.02 – 1.08) | 3.68 | <0.001 |
|  |  |  |  |  |  |
|  | 4 (243) | (Intercept) | 43.5 (23.1 – 81.7) | 11.7 | <0.001 |
|  |  | Recapture individuals | 1.05 (1.02 – 1.08 | 3.9 | <0.001 |
|  |  | Sex ratio | 0.97 (0.93 – 1.01) | -1.5 | 0.132 |
|  |  |  |  |  |  |
|  | 5 (245) | (Intercept) | 43.1 (24.3 – 76.4) | 12.9 | <0.001 |
|  |  | Recapture individuals | 1.04 (1.02 – 1.07) | 3.2 | 0.002 |
|  |  | Sex ratio | 0.97 (0.94 – 1.01) | -1.28 | 0.201 |
|  |  | Recapture individuals*Sex ratio | 0.99 (0.98 – 1.01) | -1.16 | 0.246 |
|  |  |  |  |  |  |
| Latvia | 1 (65) | (Intercept) | 31.3 (24.7 – 39.6) | 28.6 | <0.001 |
|  |  |  |  |  |  |
|  | 2 (68) | (Intercept) | 31.3 (24.7 – 39.6) | 28.6 | <0.001 |
|  |  | Sex ratio | 1.0 (0.97 – 1.03) | -0.01 | 0.990 |
|  |  |  |  |  |  |
|  | 3 (66) | (Intercept) | 30.6 (23.5 – 39.8) | 25.3 | <0.001 |
|  |  | Recapture individuals | 1.02 (0.99 – 1.05) | 1.22 | 0.221 |
|  |  |  |  |  |  |
|  | 4 (67) | (Intercept) | 30.7 (25.3 – 37.3) | 34.4 | <0.001 |
|  |  | Recapture individuals | 1.02 (1.0 – 1.04) | 1.92 | 0.054 |
|  |  | Sex ratio | 0.99 (0.96 – 1.02) | -0.93 | 0.351 |
|  |  |  |  |  |  |
|  | 5 (68) | (Intercept) | 28.6 (21.9 – 37.2) | 25.0 | <0.001 |
|  |  | Recapture individuals | 1.02 (0.99 – 1.04) | 1.5 | 0.132 |
|  |  | Sex ratio | 0.98 (0.95 – 1.01) | -1.13 | 0.26 |
|  |  | Recapture individuals*Sex ratio | 1.0 (0.99 – 1.01) | 0.76 | 0.450 |
|  |  |  |  |  |  |
